# Engineered mesenchymal stem cell-derived extracellular vesicles: A state-of-the-art multifunctional weapon against Alzheimer's disease

**DOI:** 10.7150/thno.81860

**Published:** 2023-02-05

**Authors:** Tong Yin, Yan Liu, Wenbo Ji, Jianhua Zhuang, Xiaohan Chen, Baofeng Gong, Jianjian Chu, Wendanqi Liang, Jie Gao, You Yin

**Affiliations:** 1 Department of Neurology, Second Affiliated Hospital of Naval Medical University (Shanghai Changzheng Hospital), Shanghai 200003, China.; 2 Department of Clinical Pharmacy, Xinhua Hospital; Clinical pharmacy innovation institute, Shanghai Jiao Tong University of Medicine, Shanghai 200000, China.; 3 Changhai Clinical Research Unit, Shanghai Changhai Hospital, Naval Medical University, Shanghai 200433, China.

**Keywords:** Mesenchymal stem cells, Extracellular vesicles, Alzheimer's disease, Drug delivery, Nanoparticles

## Abstract

With the increase of population aging, the number of Alzheimer's disease (AD) patients is also increasing. According to current estimates, approximately 11% of people over 65 suffer from AD, and that percentage rises to 42% among people over 85. However, no effective treatment capable of decelerating or stopping AD progression is available. Furthermore, AD-targeted drugs composed of synthetic molecules pose concerns regarding biodegradation, clearance, immune response, and neurotoxicity. Mesenchymal stem cell-derived extracellular vesicles (MSC-EVs) are essential intercellular communication mediators holding great promise as AD therapeutics owing to their biocompatibility, versatility, effortless storage, superior safety, and the ability to transport messenger and noncoding RNAs, proteins, lipids, DNAs, and other bioactive compounds derived from cells. The functionalisation and engineering strategies of MSC-EVs are highlighted (e.g. preconditioning, drug loading, surface modification, and artificial EV fabrication), which could improve AD treatment by multiple therapeutic effects, including clearing abnormal protein accumulation and achieving neuroprotection and immunomodulatory effects. Herein, this review summarises state-of-the-art strategies to engineer MSC-EVs, discusses progress in their use as AD therapeutics, presents the perspectives and challenges associated with the related clinical applications, and concludes that engineered MSC-EVs show immense potential in AD therapy.

## Introduction

Alzheimer's disease (AD) is a neurodegenerative illness responsible for up to 80% of dementia cases and is typically characterised by memory loss, cognitive impairment, behavioural abnormalities, and difficulty in performing daily activities. The World Health Organisation reports that the global number of AD patients is close to 35 million, with 10 million new cases added every year. Typical pathological changes of AD are the accumulation of β-amyloid plaques and hyperphosphorylation of Tau in neurofibrillary tangles 1, 2. Such pathological protein deposition damages the cerebral cortex and hippocampal neurons, while the release of inflammatory mediators by microglia and astrocytes during the brain immune response may in turn exacerbate the chronic neuroinflammation of AD patients 3, 4.

Traditional AD therapy relies on three cholinesterase inhibitors (donepezil, rivastigmine, and galantamine) and an uncompetitive N-methyl-D-aspartic acid receptor 2 modulator (memantine) 5-7. Recently, two new AD therapeutics have been developed, namely sodium oligomannate and Aduhelm. Sodium oligomannate, which improves the cognitive ability of mild-to-moderate AD patients, was approved in China in 2019 and is the first AD drug targeting the brain-gut axis 8. Aduhelm was approved by the Food and Drug Administration (FDA) of the United States and is the first AD drug targeting amyloid-β (Aβ) aggregates, although the related therapeutic effect remains to be discussed 9. However, the aforementioned medicines only aim to improve life quality and prolong the lifetime without preventing disease progression, and novel AD treatment strategies are therefore highly sought. One such strategy relies on mesenchymal stem cells (MSCs), which exhibit immunoregulatory and tissue regeneration abilities 10, 11. Preclinical studies revealed that MSC transplantation exerts promising therapeutic effects, such as improving hippocampal neurogenesis and synaptic activity and suppressing neuroinflammation 12. However, MSC-based therapy is limited by cell heterogeneity, immune responses, and risk of tumour formation, and primarily relies on extracellular vesicle (EV) secretion rather than on the replacement of parenchymal brain cells 13. Notably, MSCs also secrete membrane-bound EVs containing biomolecules such as growth factors and cytokines 14, 15. Exosomes are nanosized EVs with diameters of 40-160 nm that are released by a wide range of cell lines, including those found in the brain, contributing to cell-cell communication 16-19. As MSC-EVs can express the therapeutic potential of parental cells and demonstrate the merits of high penetrability through the blood-brain barrier (BBB) and low immunogenicity, they hold great promise for AD treatment 20, 21. As reported, MSC-EVs can penetrate the BBB via their active transport, probably through the traverse of microvascular endothelial cell monolayers of the brain with transiently formed interendothelial gaps 22 and target particular acceptor cells 23, 24. Rather than studying the actual brain delivery, most studies rely on therapeutic outcomes of MSC-EV treatment in AD models instead, though some researchers also directly demonstrated EV distribution 25. To date, MSC-EV therapy has achieved exceptional efficacy in many neurological disease models 10, 11, 26, 27 by reducing pathological protein accumulation, alleviating neuroinflammation and oxidative stress, and protecting the nerves 28-31. Further functionalisation of MSC-EVs has also been studied for improved therapeutic performance. Various engineering techniques, including preconditioning, drug loading, surface modification, and artificial EV fabrication, have been utilised to enhance the effectiveness of MSC-EVs as AD therapeutics. Although several reviews have discussed the potential and significance of MSC-EVs 32-34, none have presented an overview of the state-of-the-art engineering strategies for MSC-EVs and the future perspectives of these functionalised MSC-EVs for AD treatment. Therefore, the present review comprehensively discusses the methodology of MSC-EV engineering and summarises the results of MSC-EV-based AD therapy. Initially, we review the biogenesis of MSC-EVs and their isolation and purification methods, subsequently summarising the physiological functions of MSC-EVs in AD, e.g. the facilitation of Aβ degeneration, neuronal function restoration, and Aβ and Tau degradation. Subsequently, we discuss the recent developments in MSC-EV-based therapeutics with an emphasis on engineered MSC-EVs and their cargo and surface engineering techniques. Furthermore, the applications of natural and bioengineered MSC-EVs in AD treatment are discussed. Finally, we address the advantages and future challenges of MSC-EVs as bioactive nanocarriers for AD therapy, demonstrating the enormous potential of this therapy and its multiple applications.

## 2. EV biogenesis and isolation

### 2.1. Biogenesis of EVs

**Figure 1:**
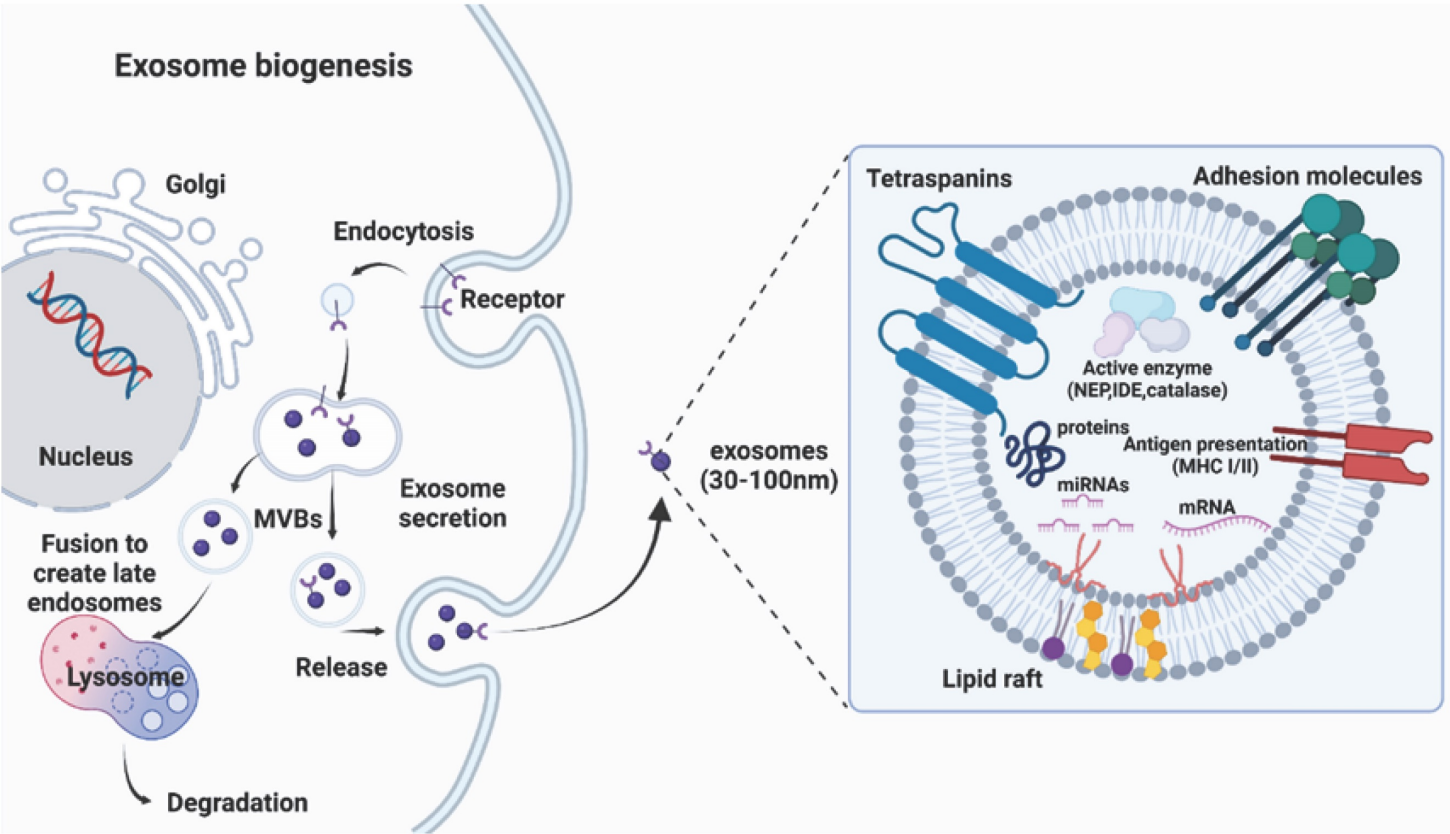
Biogenesis and structure of exosomes, which originate from multivesicular bodies containing proteins (e.g. membrane transporters) and noncoding RNAs (e.g. microRNAs, long noncoding RNAs, and circular RNAs).

There are several routes for the generation of EVs, although the exact mechanism is still unclear. There are three broad categories of EVs: exosomes (30-150 nm in diameter), microvesicles (100-1000 nm), and apoptotic bodies (100-5000 nm). Because of the ambiguous boundary of EV subclasses implied in the guidelines presented by the International Society for EVs, this review primarily focuses on well-documented nanosized EVs called exosomes, and the genesis of exosomes, rather than all EVs categories, is presented in this article (Fig. 1). Exosome genesis begins with the formation of early inward-budding endosomes, which involve the trans-Golgi network and the endoplasmic reticulum and are triggered by cell membrane endocytosis. The invasion of late endosomes affords intraluminal vesicles (ILVs), which are then converted into multivesicular bodies (MVBs) that can fuse with the plasma membrane to release ILVs and produce EVs 35 or directly fuse with lysosomes/autophagosomes 19. The fusion of MVBs with the lysosomes leads to the degradation of vesicular contents 36, while the fusion of MVBs with the plasma membrane of the cell releases vesicles via exocytosis, which are known as exosomes 37. Since exosomes are formed by double invagination processes, their topologies are identical to those in cell plasma membranes 38.

### 2.2. Isolation of EVs

The first step of downstream analyses is to fabricate high-purity and high-quality EVs in high yield. However, as EVs contain multiple components and exhibit complex heterogeneity, efficient EV isolation and enrichment methods remain highly sought after, with a summary of available techniques presented in Table 1.

Ultracentrifugation, including differential and gradient ultracentrifugation, is widely used for EV separation and purification 46. This method is useful in isolating EVs from large quantities of samples but is time-consuming 38. Concerning gradient ultracentrifugation, EVs remain in the density equilibrium region and can therefore be separated from impurities 47. However, repeated centrifugation and high centrifugal forces can irreversibly damage the isolated vesicles 48. An innovative new technique for separating EVs from large samples is size-exclusion chromatography 49, relying on filtration through a column filled with a stationary phase containing porous beads of comparable size to the particles of interest 50. This method offers the advantages of time savings, low cost, and repeatability but suffers from poor EV recovery and purity. Ultrafiltration, another size-based separation method, relies on EV interception by membranes with different pore sizes and offers the benefits of being efficient, economical, and easy and allows batch processing. However, ultrafiltration membranes possess narrow pores and are easily clogged when used to separate biological samples, while the excessive pressures used for ultrafiltration may induce EV deformation 51. Additionally, EVs can be isolated using commercial kits that rely on polymer-based coprecipitation. Although this method eliminates the use of ultracentrifugation, which is costly and damages vesicles, it suffers from the potential coprecipitation of other macromolecules 38. In general, the abovementioned methods exploit the physical properties of EVs and are not particularly specific.

The specificity of EV isolation can be improved using affinity-based capture strategies that utilise the high affinities of specific exosomal markers of their ligands 52. Considering the similar morphologies, overlapping size ranges, and inherent heterogeneity of EVs, comprehensive characterisation and consistent purification processes that allow EV subtype isolation are lacking. There are advantages and disadvantages of each method, and several methods can be combined to maximise EV enrichment. Since of the main obstacles to the clinical use of EVs is the reproducibility of their extraction, optimized and standardized methods also need to be established.

After isolation, EVs are mostly characterized by size measurement (nanoparticle tracking analysis [NTA], dynamic light scattering, etc.), morphology evaluation (electron microscopy or atomic force microscopy) or assessment of enriched protein expression (western blot, enzyme-linked immunosorbent assay, etc.) 53. The most used surface markers to identify MSC-EVs are CD73, CD105 and CD90 54. However, the surface components of EVs derived from different MSCs are affected by their parent cells, so proteomics for MSC-EVs from different tissues needs further study (Table 2). The methodology for EV storage is rarely reported, although it affects both morphology and physicochemical parameters of EV simultaneously. As of now, 80°C seems to be the most promising storage temperature for EVs. However, the cost and transportation challenges of applying this mode may limit its application. It is therefore possible to enhance EV storage stability by using alternatives such as lyophilization and addition of additives 55. The administration of MSC-EVs in AD treatment include localized administration (eg. dorsal hippocampus injection 56) and systemic administration (eg. intravenous injection 57, intranasal administration 58). Due to the BBB permeability of MSC-EVs, systemic administration has the advantage of minimally invasive over other administration routes.

## 3. Brain delivery of MSC-EVs

The BBB controls the entry of nutrients from the vascular system into the extracellular fluid of the central nervous system (CNS) and limits the passage of harmful exogenous molecules to maintain homeostasis, which severely limits the therapeutic efficacy of drugs against CNS diseases including AD 66. Advantageously, MSC-EVs, which contain small-molecule nucleic acids, proteins, and other cell growth regulators, can penetrate the BBB to exert therapeutic effects in the brain and have therefore been adopted in a wide range of studies on different diseases, such as AD (Table 3) 67-71.

One of the most remarkable characteristics of MSC-EVs is their capacity to circulate in the bloodstream and cross the BBB. Although it is unclear how natural MSC-EVs cross the BBB, researchers have found that MSCs crossed the brain microvascular endothelial cell (BMEC) monolayers through transient intercellular gaps formed between BMECs 72. Transmigration of MSCs across the endothelium in peripheral tissues is mediated by integrin-mediated adhesion or matrix metalloproteinase-dependent extracellular matrix degradation 73, 74. Because the membrane of EVs derived from MSCs shares similar features with MSCs, these findings may offer clues for further investigation into the exact mechanism underlying BBB penetration by MSC-EVs. Moreover, as BMECs cannot represent the function of the complete BBB, a quantitative and qualitative method is needed for monitoring MSC-EV delivery *in vivo* to the brain 75.

Apart from the traditional intravenous (IV) delivery strategy, nasal delivery has also been studied as a non-invasive method to send MSC-EVs directly to the brain 58, 76, and the olfactory mucosa pathway in the olfactory region is the most essential route to the brain 77. Intranasal administration of PKH-26 labelled MSC-EVs mainly reached neurons, compared with microglia and astrocytes 78, while another study observed a higher absorption by microglia and neurons in the hippocampus but not by astrocytes 58. In addition, EVs labelled with C5 Maleimide-Alexa 594 were found in hippocampal neurons 24 hours after their IV injection 31. Five hours after IV injection, MSC-EVs labelled with DiI were detected in the brain cortex, hippocampus, lungs, and spleen 57.

It is noteworthy that the lipid dyes used in MSC-EV studies have the limitations of non-specific marking and formation of aggregates from unbound dye 79, which can result in artefactual results and erroneous conclusions. Therefore, the derivation of MSC-EV delivery requires experimental methods such as advanced imaging techniques and rigorous controls 79.

## 4. Physiological functions of MSC-EVs in AD models

Initially, MSC therapies were demonstrated in bone and cartilage to induce tissue repair and regeneration 80. Several MSC therapies became available soon after, including treatments for brain diseases 81. MSC therapy studies conducted in mice and humans initially confirmed positive results, with clinical trials expected soon 82, 83. Despite these positive results, negative effects soon occurred, including immunological rejection and cancerous growth due to the size of the cells and the risk of embolism complications 84. These unexpected inconveniences lead to rapid reductions in MSC therapies, followed by the withdrawal of initial clinical trials. The discovery that MSC therapies had serious problems led to studies on their secreted vesicles 81, 85. Several curative effects of MSCs have been recapitulated by MSC-EVs, which also leads to unexpected benefits, such as higher safety and tissue penetration 86. Moreover, MSC-EVs are incapable of self-replicating, which prevents many risks of MSC therapy. In comparison with cell-based approaches, MSC-EV therapy demonstrated favourable effects like activating the immune system and prolonging the therapeutic effects 87.

**Figure 2:**
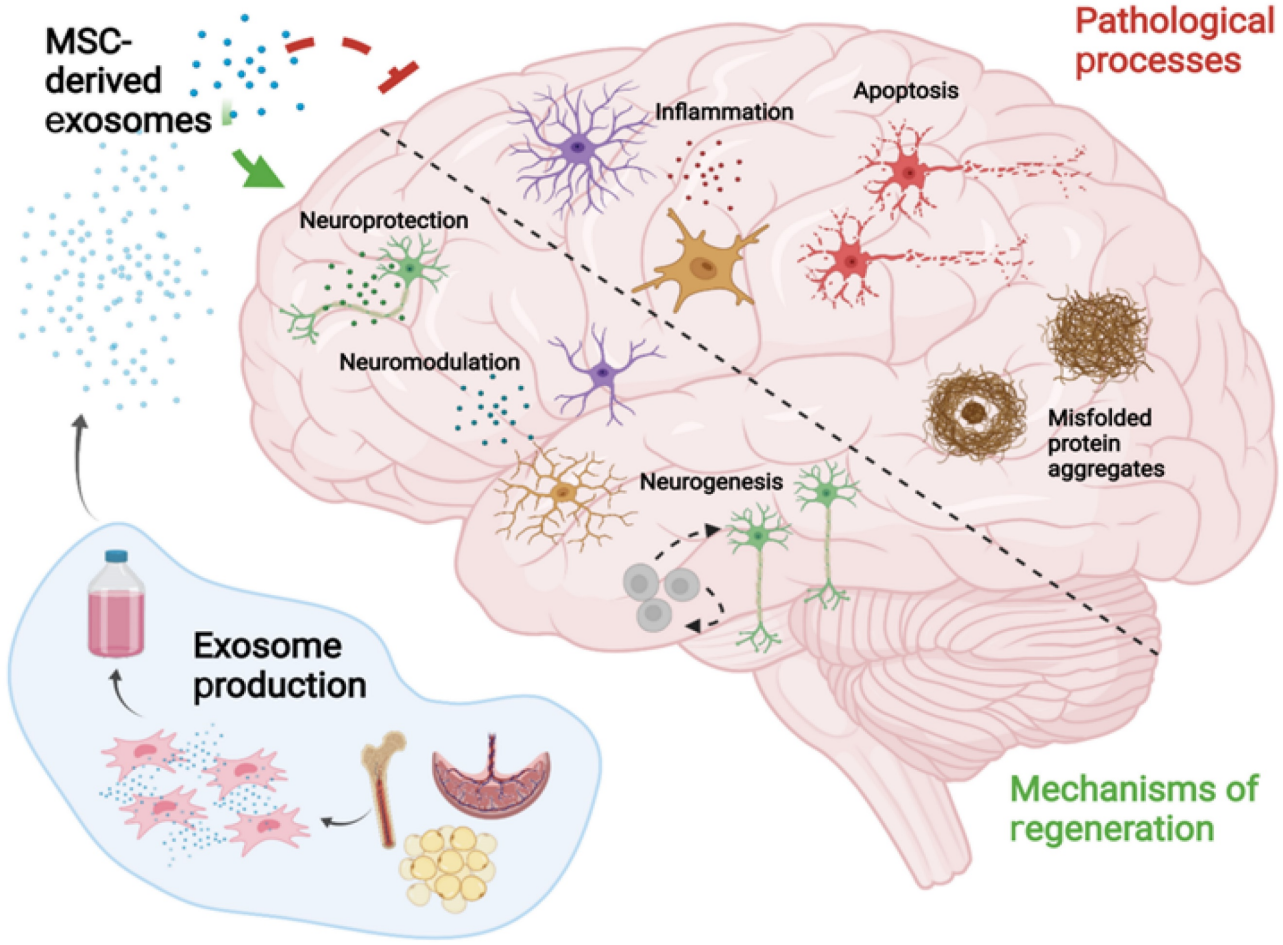
Main pathological processes of AD and the key mechanisms by which MSC-EVs mitigate the related pathogenesis and induce nerve regeneration.

MSC-EVs were observed to improve pathological changes and cognition in AD mice by clearing abnormal protein accumulation 14, 29, 88, achieving neuroprotection 32, 58, 89, relieving inflammation 90, 91, and alleviating oxidative stress 31, 92, 93 (Fig. 2). MSC-EVs exhibit the homing ability and can target pathological regions in AD models, thus representing promising drug delivery platforms for AD treatment 94. Nevertheless, the EVs that do not naturally express targeting ligands have the least penetrating capacity, and thus the functionalisation of MSC-EVs seems to become a solution, which has been discussed in 5.1.3.

Apart from MSC-EVs, AD has been studied using EVs derived from other cell lines. In the brain, astrocytes, which are the most abundant glial cells, are used to produce exosomes with ultrasound stimulation 95. The result indicated that exosomes secreted by astrocytes can reduce *in vitro* toxicity and plaques caused by Aβ in AD mice. To manage the neuroinflammation linked to AD, yeast cell wall vesicles loaded with sitagliptin, a medication having an anti-inflammatory action, was created 96. It was shown that the optimised vesicles were less toxic than the sitagliptin due to their spherical shape and negative surface charge.

In this review, we focus on MSC-EVs for the unique characteristics inherited from their parent cells and may perform superior functions to EVs derived from other cells, which has been discussed in section 8.1.

### 4.1. Clearance of abnormal protein accumulation by MSC-EVs

Pathological proteins, including Aβ plaques and hyperphosphorylated tau, which cause synaptic disruption and neuronal degeneration, are the key elements in the pathology of AD, and the removal of these abnormal proteins will significantly benefit AD treatment 101. Notably, natural MSC-EVs have been discovered to have the potential for pathological protein clearance through modulating autophagy and regulating the levels of critical proteins in AD development with inherited proteins and microRNAs (miRs). miR is a set of non-coding RNA of 22-24 nucleotides long that can be found in EVs and attaches to the 3′ untranslated region of mRNA to silence the mRNA transcript 102. Recent studies have investigated miRs associated with various pathways in AD patients 103, 104.

Recent research published in *Nature Neuroscience* has revealed that lysosome dysfunction that causes autophagy failure has a close correlation with AD pathology and Aβ deposition, that is, autolysosome acidification declines in neurons before extracellular amyloid deposition, associated with a significant decrease in v-ATPase activity and selective accumulation of Aβ/Aβ precursor protein (APP) within enlarged de-acidified autolysosomes 105. In addition, autophagy activation was proven to be effective in Aβ plaque scavenging, indicating that autophagy activation represents a promising strategy against AD through clearance of abnormal protein accumulation 105. It is noteworthy that MSCs and MSC-EVs show the intrinsic ability to activate autophagy to increase the reduction of abnormal protein, resulting in the remarkable amelioration of AD 106, 107.

In addition to the intrinsic ability to activate autophagy for the clearance of abnormal protein, MSC-EVs could also regulate the expression of a key protein that could clear abnormal protein. For example, researchers also found that MSC-EVs exert a therapeutic role in AD treatment through the regulation of sphingosine 1-phosphate (S1P) expression, which can stimulate autophagy, protect neurons, regulate the inflammatory response of glial cells, and antagonise apoptosis to inhibit the pathological changes induced by AD 108. S1P receptors are expressed throughout the body and play key roles in angiogenesis, neurogenesis, immune cell transportation, endothelial barrier functioning, and vascular tone regulation 109. It has been reported that MSC-EVs improve spatial cognition in AD mice and promote S1P and sphingosine kinase (SphK) 1 expression, which catalyses the phosphorylation of sphingosine to S1P 98. Furthermore, MSC-EVs reduce amyloid levels and enhance the expression of the anti-neuronal core antigen of APP/PS1 mice, decreasing the concentrations of Aβ oligomers (AβOs), β-site APP-cleaving enzyme (BACE) and presenilin 1 and promoting the expression of neprilysin (NEP). In conclusion, the abovementioned studies found that MSC-EVs improve memory restoration in AD mice through the activation of the SphK/S1P signalling pathway and reduce Aβ deposition through various mechanisms, including autophagy modulation. In addition, NEP is a completely membrane-bound metallopeptidase that degrades neuropeptides and amyloid proteins and is therefore a potential target for AD treatment 110. Importantly, NEP is known to exist on MSC-EVs, and its role in reducing Aβ deposition has been evaluated *in vivo* and *in vitro* 65, 100, 111.

MSC-EVs have also been discovered to have the potential for pathological protein clearance through their inherited miRs. APP proteolysis begins with cleavage by β-secretase, also denoted BACE, which makes BACE1 inhibitors promising AD therapeutics 112. MiRs are noncoding RNAs closely related to AD pathogenesis and demonstrate enormous potential as therapeutic biomarkers 113. Recent studies have demonstrated that BACE1 regulates AD progression via miRs 86, 114. Sha et al. 97 discovered that miR-29c-3p delivered to neurons by bone marrow MSC-EVs (BMSC-EVs) inhibits BACE1 expression and activates the Wnt/β-catenin pathway to downregulate the level of BACE1^115^ and thereby exert a therapeutic effect on AD. Jeong et al. demonstrated that compared with Aβ injection groups, human umbilical cord-derived mesenchymal stem cell (hUCMSC) groups exhibited a significant reduction in amyloid plaque and BACE1 and an increase in neurogenesis 65.

Overall, lysosome dysfunction, autophagy failure and accumulation of abnormal protein are the main characteristics of AD. MSC-EVs can effectively restore the autophagy function and accelerate the clearance of abnormal protein through multiple mechanisms, and finally provoke distinguished anti-AD effects, thus representing a promising strategy against AD.

### 4.2. Immunomodulatory effects of MSC-EVs

Neuroinflammation contributes significantly to AD pathogenesis, which is mainly characterised by overactive microglia, morphological and functional alterations in neurons, and upregulated inflammatory factors 116. During the early stages of AD, activated microglia are primarily characterized by their neuroprotective and anti-inflammatory M2 phenotype. As AD progresses, hyperactivation of microglia becomes detrimental and pro-inflammatory, resulting in the M1 phenotype 117. BMSC-EVs were reported to contain miR-146a, which contributes to microglial polarisation and is notably overexpressed in proinflammatory microglia (M1 type) 118. In addition, a high level of miR-146a is detected in the brains of AD patients, and its expression is negatively regulated by nuclear factor-κB, therefore representing a promising therapeutic 81. Nakano et al. reported that miR-146a from BMSC-EVs is taken up by astrocytes and inhibits inflammation in AD mice models 81. The treatment of AD model mice with BMSCs was shown to improve synaptic density, alleviate inflammation within astrocytes, and reduce the M1 microglia ratio. The same function was found in EVs secreted by adipose tissue-derived mesenchymal stem cells 119.

IL-10 is a cytokine that suppresses immune responses in peripheral immunity. Studies aimed at understanding the antiphlogistic mechanisms of MSCs discovered that IL-10 plays an important role in macrophage phenotype switching [from the M1 (inflammatory) type to the M2 (anti-inflammatory) type] triggering MSC-induced repair 120. Thus, IL-10 is essential to regulate aberrant immune activation and signalling associated with AD. Feng et al. discovered that MSC-EVs can mitigate trained immune responses in the CNS and demonstrated the important role of IL-10 in this process 88. The development of taste disorders results from the activation of inflammatory pathways 121. Exosomes derived from BMSCs can reduce the destructive changes caused by AD in circumvallate papilla taste buds 91.

MSC-EVs also exert a therapeutic effect on AD by reducing oxidative stress, which is vital for the pathogenesis of cognitive deficits and the development of neurological injuries 122. Overexpressed or dysregulated proinflammatory cytokines (iNOS) contribute to AD pathology 123. Wang et al. observed that exogenous Aβ-induced iNOS mRNA and protein expression was reduced by MSC-EVs, which exerted beneficial effects on APP/PS1 mice 92. Currently, an increasing amount of evidence indicates that neurotrophic factor (NF)-E2-related factor like-2 (Nrf2) participate in the redox state and anti-inflammatory effects in neurodegeneration 124. Notably, the Nrf2 signalling pathway increases antioxidant gene expression, inhibits microglia-mediated inflammation, and improves mitochondrial functioning in diseases of the nervous system, which indicates that Nrf2 activation is an innovative therapeutic approach to target AD development 125. In APP/PS1 mice, the Nrf2 defence system participates in reducing neuronal deficits caused by MSC-EVs 31.

AD pathophysiology is affected by glial changes in morphology, function, and gene expression caused by AβO that lead to synaptotoxicity and neuronal death 126, 127. Godoy et al. found that MSC-EVs naturally contain and carry endogenous catalase and provide protection against oxidative stress by delivering this enzyme to neurons and preventing synaptic loss in neurons exposed to AβOs 93.

In general, the above studies show that MSC-EVs alleviate inflammation through various mechanisms (e.g. microglial phenotype switching, inflammatory cytokine regulation, and oxidative stress reduction) and thus hold great promise for AD therapy.

### 4.3. Neuroprotective effects of MSC-EVs

MSC-EVs can improve AD symptoms by exerting neuroprotective effects and promoting neurogenesis. For example, miR-223 delivered by MSC-EVs protects neurons from apoptosis by activating the phosphatase and tensin homolog-phosphatidylinositol-3-kinase (PTEN-PI3K/Akt) pathway 28. As astrocytes are the key cells that form synapses, recovery of astrocyte function may lead to synaptic occurrence and cognitive impairment. Nakano et al. discovered that the intracerebroventricular injection of BMSC-EVs containing miR-146a ameliorates cognitive impairment in AD mice by reducing astrocyte inflammation and improving synaptic development 81. Similarly, Chen et al. found that in SH-SY5Y human neuroblastoma cells, MSC-EVs increase gene expression associated with synaptic plasticity and memory, including Homer1, GluR1, GluR2, NR2A, NR2B, Syp, and brain-derived NF (BDNF exon IV) 32. Treatment with MSC-EVs can also be used to regulate the neuronal and astrocyte phases of AD mice 32. Wang et al. reported that MSC-EV therapy restores the alterations of dendritic spines, calcium oscillations, abnormal action potential, and mitochondrial changes in AD models, highlighting the therapeutic efficacy of MSC-EVs toward AD 31. Additionally, MSC-EV therapy through intranasal delivery 76 and cerebrospinal fluid (CSF) exchange 90 increases the neuronal density in the brain, which is typically related to reduced hippocampal shrinkage. Overall, these studies indicate that MSC-EVs can protect neurons and astrocytes and accelerate nerve regeneration in AD mice by recovering functionality and neural cells, thus holding great promise for AD treatment.

## 5. Engineered MSC-EVs for AD therapy

### 5.1. EV engineering techniques

**Figure 3:**
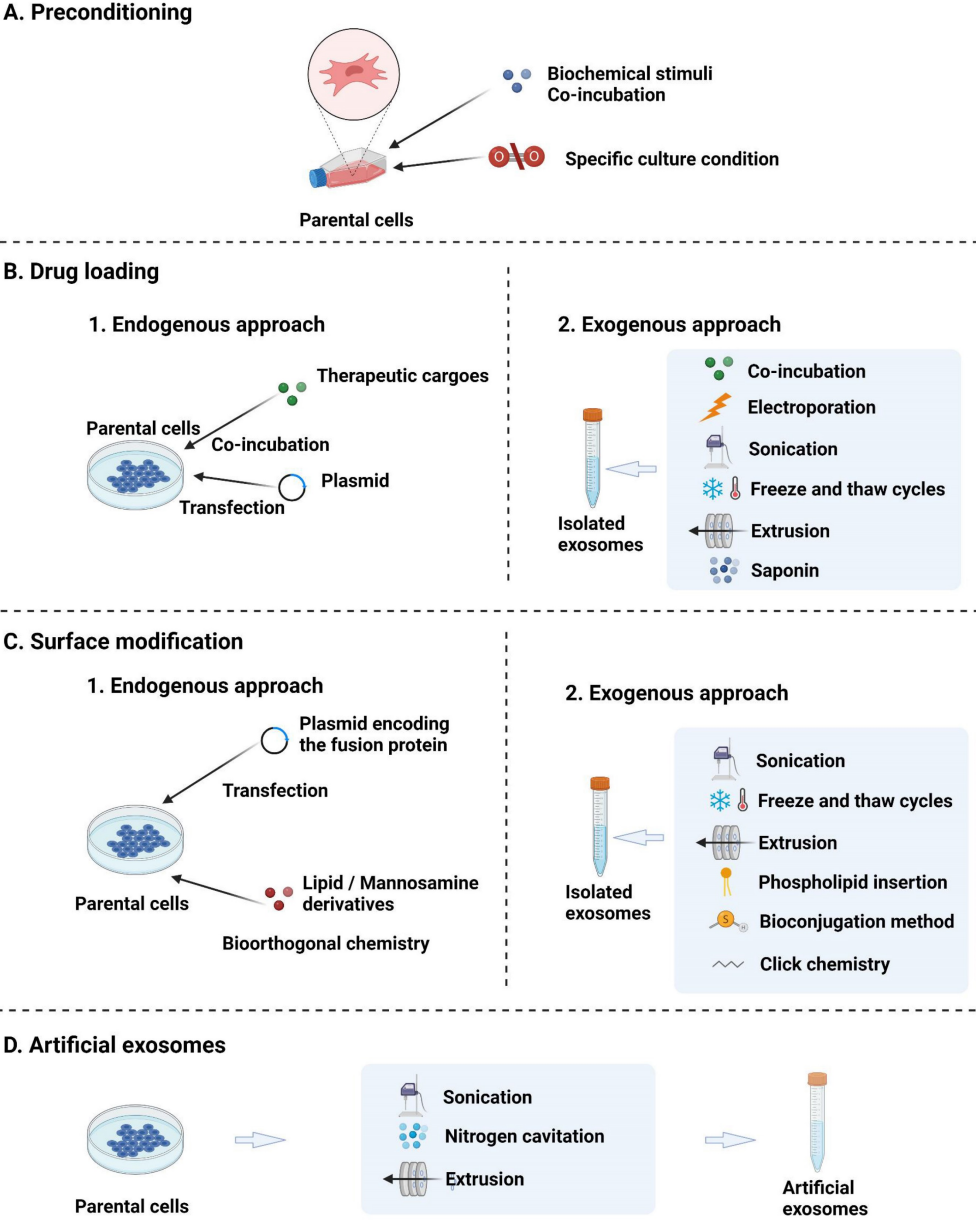
Strategies for the engineering of EVs: (A) preconditioning of parental cells to enhance the inherent treatment effect; (B) loading of therapeutic cargoes; (C) surface modification; and (D) fabrication of artificial EVs through the top-down disruption of parent cell membranes.

Although MSC-EVs can be of therapeutic value for AD, their application is still hindered by several drawbacks, including low targeting efficiency, nonuniform treatment results, and low production yield 128. Therefore, advanced engineering techniques are needed to functionalise MSC-EVs and thus increase their efficiency and potency as AD therapeutics. These techniques include parental cell preconditioning to enhance the inherent treatment effect, therapeutic cargo loading into MSC-EVs, MSC-EV surface modification to enhance targeting, and artificial MSC-EVs to improve productivity (Fig. 3).

#### 5.1.1. Preconditioning

Preconditioning includes parent cell manipulations using specific culturing conditions, such as hypoxia, 3D culturing, and serum deprivation. Biochemical stimuli, including lipopolysaccharides, nitric oxide, and proinflammatory cytokines and exogenous genes such as plasmid DNA and miRNAs, can also be introduced into the culturing environment 129-131.

Preconditioning-based strategies are often used to enhance MSC functional characteristics before transplantation through culturing environment modification. EV generation by cellular metabolic processes is partly responsible for the genetic and phenotypic characteristics of the cells used for MSC-EV production, which can be altered by extrinsic stimulation 132. Nevertheless, considering various physiological conditions of MSCs, the optimisation of preconditioning approaches needs further research. As preconditioning can stress parental cells, it is necessary to investigate the generated contents in EVs that might negatively impact therapeutic results.

#### 5.1.2. Drug loading

As part of the cargo delivery system, EVs can be used to treat recipient cells by releasing both naturally formed contents and loaded therapeutics such as chemicals, proteins, peptides, and nucleic acids. Drug loading strategies can be divided into two categories: endogenous (drug-loaded EVs are collected after parental cell pretreatment using various approaches) and exogenous (exogenous drugs are loaded into isolated EVs) 133.

1) In the endogenous approach, therapeutic cargoes are loaded into EVs using the intrinsic cellular cargo transfer system and are packed in the secreted EVs 134. The endogenous approach may be preferred to the exogenous approach, allowing EVs to be loaded with cargoes at the cellular level and facilitating small RNA expression induced by vector transfection 135. However, the dependence on parent cell physiology may not allow for customised modification and result in uneven cargo levels in EVs 135. The endogenous approach can be subdivided into two methods. In the first method, drugs are cocultured with parent cells and are naturally present in the secreted EVs. However, the achieved transfection efficiency is low, and this method is therefore mainly used for chemical drugs with low cytotoxicity 136. In the second strategy, parent cells are transfected with chemical methods such as liposome transfection to carry drugs, although the transfection efficiency is low 137. This method is mainly used for gene drugs, which can be transfected into parental cells to overexpress the elements of interest that are subsequently transferred into EVs and function within the target cells 138.

2) Exogenous strategies allow EVs to be loaded with specific drugs with better control over cargo loading and can be categorised as follows: 1) Coculturing of drugs with extracted EVs (for chemical drugs). The incubation of cargoes with EVs is a simple method, but the loading efficiency requires improvement, as the scope of loaded drugs is often limited to hydrophobic compounds 139. 2) Transfer of drugs into EVs using physical strategies. A common physical approach to drug loading is electroporation, wherein charges are loaded into EVs using weak current pulses, and the phospholipid bilayer is broken down to form a recoverable hole that facilitates drug loading 140. Other physical strategies, such as hypotonic dialysis, saponin incubation, extrusion, sonication, pH neutralisation, and freeze-thaw cycling, have also been used to exogenously load therapeutic drugs into natural EVs 141. 3) Direct transfer of drugs into EVs by chemical (e.g. liposome) transfection methods. In this case, transfection reagents or permeabilisers are used to facilitate cargo entry into EVs without damaging the lipid bilayer structure. For example, acoustofluidics has been demonstrated as an effective and fast way of loading drugs into EVs 142. In general, during postloading, EV aggregation, membrane damage, or the elicitation of inflammation should be cautiously avoided.

All the above-mentioned methods have been used to load cargo into EVs. However, as the drug loading efficiency may depend on the method used 140, the related techniques need to be optimised for maximal drug loading efficiency.

#### 5.1.3. Surface modification

The surface modification allows natural EVs to be imbued with unique functionality, as exemplified by the targeting of peptides or sites for chemical modification via genetic manipulation 143. Similar to cargo loading, surface functionalisation can be categorised into endogenous and exogenous functionalisation. 1) Endogenous approaches allow the EV surface to be genetically engineered to bear specific peptides or ligands by transfecting cells with vectors encoding these molecules 144. Moreover, EVs can be endogenously modified using bioorthogonal chemistry, which is characterised by high efficiency and reproducibility. Even though endogenous approaches maintain important EV functionality and integrity, they suffer from heterogeneity in the secreted EV population and the complexity of the purification process used to separate functionalised EVs from native EVs.

2) In contrast to endogenous approaches, exogenous surface modification is performed directly on the EV membrane by physical and chemical means. Physical methods include extrusion, sonication, and freeze-thaw cycling 145 and allow for reagent-free functionalisation but may result in internal cargo loss. Chemical approaches include a series of facile click chemistry processes to covalently conjugate EV lipid or protein constructs with different linker groups to achieve various functionalities, as exemplified by thiol-maleimide 146 and azide-alkyne cycloadditions 147. Regardless of the method used for EV functionalisation, it is essential to investigate whether surface modification affects the functionality, integrity, and therapeutic potential of EVs.

#### 5.1.4. Artificial EVs

MSC-EV usage as therapeutics demands processes that afford EVs in high yields and are suitable for use in clinical settings. Unlike natural EVs, artificial EVs are usually produced by disintegrating the cell membrane with top-down strategies, including extrusion, microfluidic technologies, nitrogen cavitation, and sonication, and are therefore obtained in much higher yields 148. Several intracellular elements of parental cells can be preserved in artificial EVs using these processes 149. Notably, artificial EVs can contain up to twice as much RNA and protein as natural EVs 128 and can be alternatively loaded with therapeutic molecules. For example, drugs can be added into artificial EVs during the shearing process or by shearing cells preloaded with cargoes of interest 150. Similar to EVs, these artificial molecules can also be loaded via electroporation and vector infection 151, 152. Considering their similarity to their endogenous counterparts and, more importantly, their ability to bypass the time-consuming and labour-intensive isolation procedures of natural EVs, artificial EVs represent a promising alternative to natural EVs 153. Because artificial EVs are a new field, there is a lack of information from both preclinical and clinical studies for AD therapy, further research on these nanocarriers in AD treatment is needed. There are still several difficulties in realising the clinical benefits of artificial EVs, including 1) contamination with other organelles and nuclei during the purification process, 2) low coating efficiency due to the limited quality control, and 3) the ratio of cell membranes if fused membranes are used 154. Thus, for artificial EVs, standard operating procedures, quantification of cell membranes on cargoes, and the composition of hybrid cell membranes are required to be clarified for their further application in AD treatment.

### 5.2. Applications of bioengineered MSC-EVs in AD therapy

Previously, we discussed recent technological developments in the engineering of therapeutic EVs. As the use of artificial MSC-EVs in AD treatment has not yet been reported, sections 5.2.1-5.2.3 present a review of the current applications of preconditioned, drug-loaded, and surface-modified MSC-EVs in AD therapy (Table 4).

#### 5.2.1. Preconditioned MSC-EVs for AD therapy

**Figure 4:**
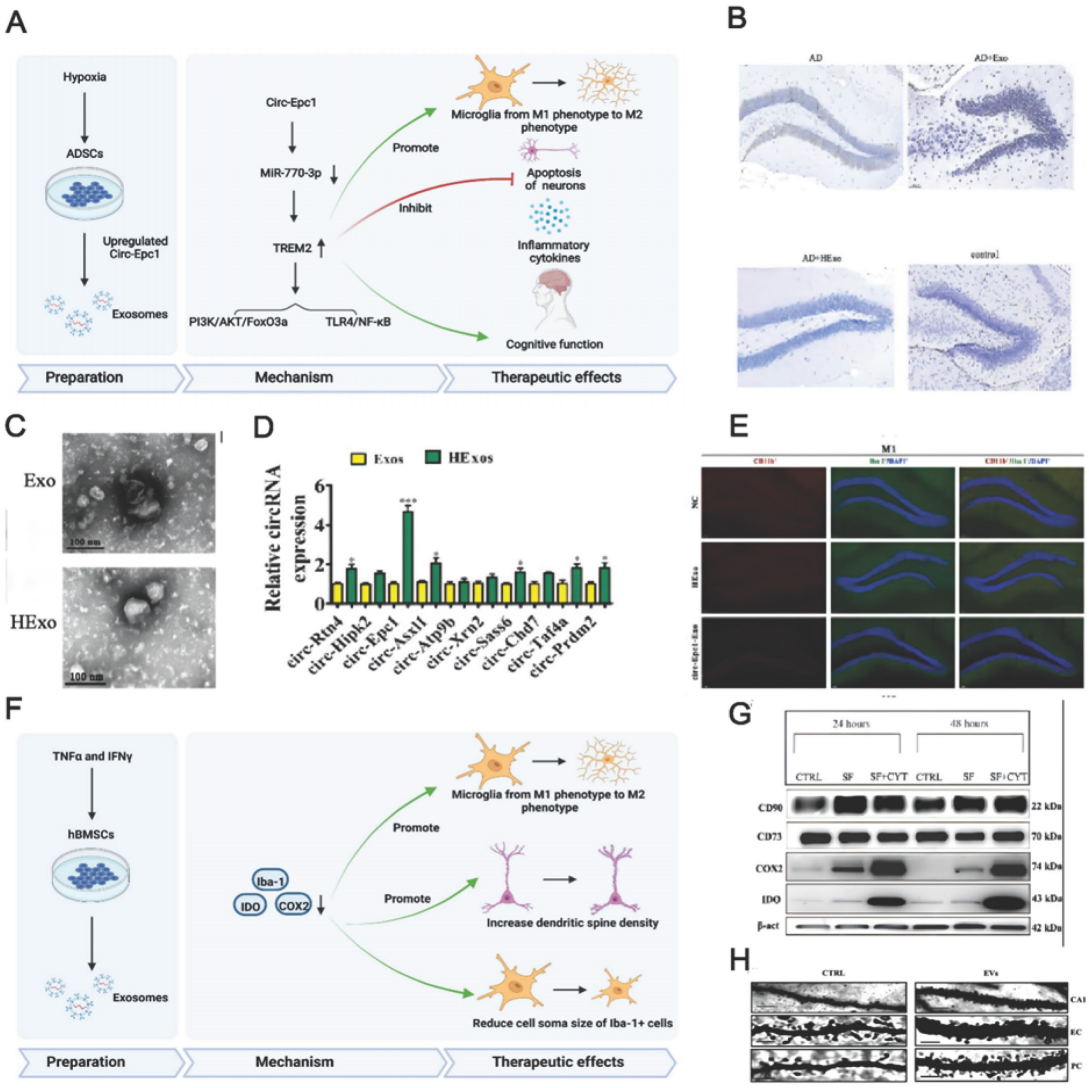
(A) Schematics of the use of hypoxia-preconditioned MSCs-derived exosomes in AD therapy. (B) Hippocampal neuron apoptosis detection by terminal deoxynucleotidyl transferase nick end labelling. (C) ADSC-exosome morphology under a transmission electron microscope. Scale bar: 100 nm. (D) Reverse transcription-quantitative polymerase chain reaction detection of circRNAs. (E) Detecting macrophage polarisation with immunofluorescence. Reproduced from ref. 156. Copyright © 2022 Haining Liu et al. (F) Schematics of the use of TNFα and IFNγ-preconditioned MSC-derived exosomes in AD therapy. (G) Typical stemness and immunoregulatory markers expressed by the MSCs (CTRL: control; SF: serum‐free; CYT: cytokines, TNFα and interferon gamma). (H) Photomicrographs of Golgi‐Cox-stained dendritic segments. Reproduced from ref. 58. Copyright © 2022 Morris Losurdo et al.

Preconditioned MSC-EVs can be used for AD treatment and are most prepared using hypoxic culturing and preconditioning with proinflammatory cytokines. Having MSCs accustomed to the hypoxic environment in advance may help them cope with the barren environment *in vivo*. High levels of hypoxia-inducible factor (HIF-1α), ROS (reactive oxygen species), and anti-inflammatory cytokines may enhance the neuroprotective ability of normoxic- and hypoxic-cultured MSCs 158. Additionally, hypoxia participates in AD development, and intensive hypoxic training may prevent neuronal loss associated with AD and promote cognitive functions 159. Using adipose tissue-derived stem cells (ADSCs) pretreated with hypoxia, Liu et al. revealed that ADSC-derived exosomes improved cognition by reducing neuronal damage in AD models 156. Circular RNA-Epc1 (Circ-Epc1), which is upregulated in these exosomes, was responsible for improving cognitive functions, protecting neurons, and shifting microglial M1/M2 polarisation in exosome-treated APP/PS1 mice (Fig. 4A-E). Similarly, Cui et al. found that miR-21 overexpression in hypoxia-preconditioned MSC-EVs not only decreased Aβ deposition but also reduced proinflammatory factors, including TNF-α (tumor necrosis factor-α) and IL-1β (interleukin-1β) 57. In addition, EVs derived from MSCs preconditioned with hypoxia improved cognitive function by preventing synaptic malfunction and alleviating inflammation in AD mice.

The immunoregulatory capacity of MSC-EVs can also be increased by preconditioning with proinflammatory cytokines. Losurdo et al. discovered that EVs derived from TNF-α and INFγ -preconditioned MSCs decreased microglial activation and increased dendritic spine density in AD to promote immunomodulatory and neuroprotective effects (Fig. 4F-H) 58. Paracrine mechanisms are activated when infused MSCs are exposed to an injured environment by releasing bioactive factors in their secretome.

**Figure 5:**
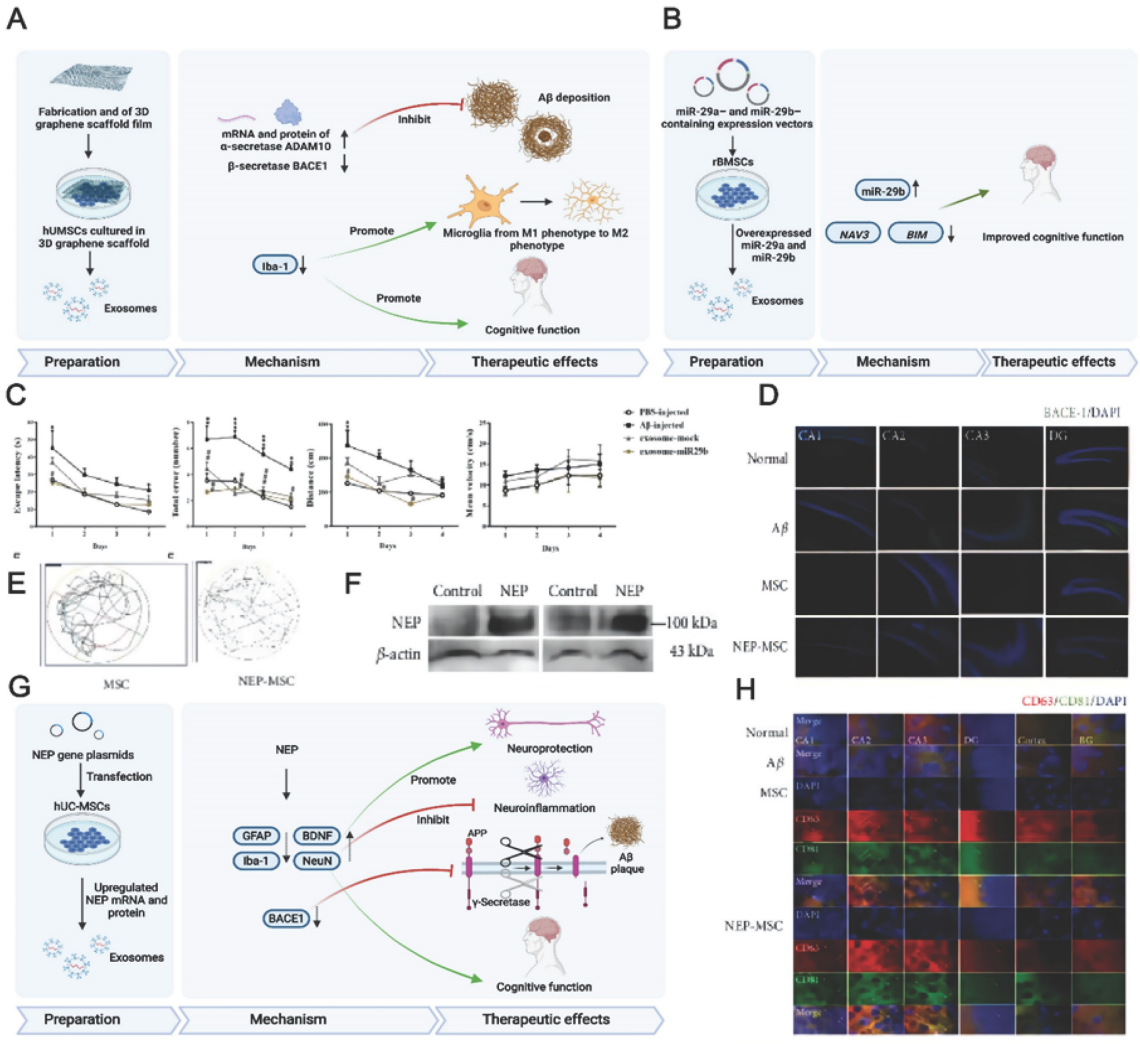
(A) Schematics of the preparation, action mechanism, and therapeutic effects of 3D-cultured MSC-EVs in AD therapy. Ref. 157. (B) Schematics of the use of miR-29-loaded MSC-derived exosomes in AD therapy. (C) The escape latency, total errors, distance, and mean velocity of AD mice after transplantation of the engineered MSC-EVs containing miR-29b. Reproduced from ref. 56. Copyright © 2020 Yavar Jahangard et al. (D) BACE-1 expression in the hippocampus. Reproduced from ref. 65. Copyright © 2021 HyeJu Jeong et al. (E) The swimming path of the mice in Morris water maze test. (F) The change of NEP level in hUCMSC-EVs. (G) Schematics of the preparation, action mechanism, and therapeutic effects of NEP mRNA- and protein-loaded MSC-EVs in AD therapy. (H) The colocalisation of CD63 and CD81-positive hUCMSC-EVs in the hippocampus. Reproduced from ref. 65. Copyright © 2021 HyeJu Jeong et al.

A previous study demonstrated that using a secretome collected from MSCs preconditioned *in vitro* in an AD environment led to sustained memory recovery, neuropathology repair, increased neuronal density, and reduced hippocampal shrinkage in APP/PS1 mice while increasing their lifespan 76. Growing evidence has demonstrated that 3D cultured cells can produce a more realistic physiology. For example, treatment with 3D cultured exosomes was shown to upregulate the expression of β-secretase and thus reduce Aβ production *in vivo* and *in vitro* (Fig. 5A) 157.

#### 5.2.2. Drug-loaded MSC-EVs for AD therapy

MSC-EVs can be loaded with diverse therapeutics, including nucleic acids, proteins, and drugs. AD patients showed reduced RNA-29 (microRNA-29) expression, which contributed to AD pathogenesis. Jahangard et al. revealed that treatment with miR-29b-loaded BMSC-EVs helps to restore the learning function and prevent memory loss in an Aβ-induced AD model (Fig. 5B-C) 56. In this study, BMSCs were transfected with vectors containing miR-29b to subsequently derive miR-29b-containing exosomes. The underlying therapeutic effect of miR-29b against AD is based on the downregulation of NAV3 (neuron navigator), β-site amyloid precursor protein cleaving enzyme 1 (BACE1) and Bcl-2 interacting mediator of cell death (BIM) expression. Similarly, exosomes derived from miR-22-transfected ADMSCs exhibited therapeutic effects on both P12 cells and AD mice 155. EV treatment also resulted in the acceleration of neuron regeneration, the alleviation of neuroinflammation, and the downregulation of inflammatory factors while improving the behavioural and memory abilities of AD mice.

Proteins as important regulator molecules, play a critical role in various disease therapies. A growing number of studies show that genetically engineered MSC-EVs containing therapeutic proteins were used in disease treatments 160-162. For example, in the context of AD, Xu F et al. infected MSCs with lentivirus encoding the Src homology 2 domain-containing protein tyrosine phosphatase-2 (SHP2) gene to obtain MSC-EVs with high level of SHP2 162*.* As a result, mitophagy is significantly induced by MSC-EVs-SHP2, which ameliorates mitochondrial damage-mediated apoptosis and NLRP3 activation in neuronal cells.

In the brain, NEP plays an important role in Aβ clearance 163. Jeong et al. overexpressed NEP in human umbilical cord tissue-derived MSCs (hUCMSCs) by transfection 65, revealing that NEP plasmid transfection into hUCMSCs increases NEP gene expression and protein levels. The transplantation of NEP-enhanced hUCMSCs into AD mice improved their memory and cognitive function. The underlying therapeutic mechanism of NEP-enhanced hUCMSCs includes the suppression of Aβ accumulation and BACE1 expression, the regeneration of brain-derived BDNF activity, neurogenesis promotion, and increased NEP expression. The therapeutic efficacy of hUCMSCs was attributed to MSC-EVs rather than to MSC penetration, which shows that MSC-EVs are promising for AD treatment (Fig. 5D-H).

#### 5.2.3. Surface-modified MSC-EVs for AD therapy

**Figure 6:**
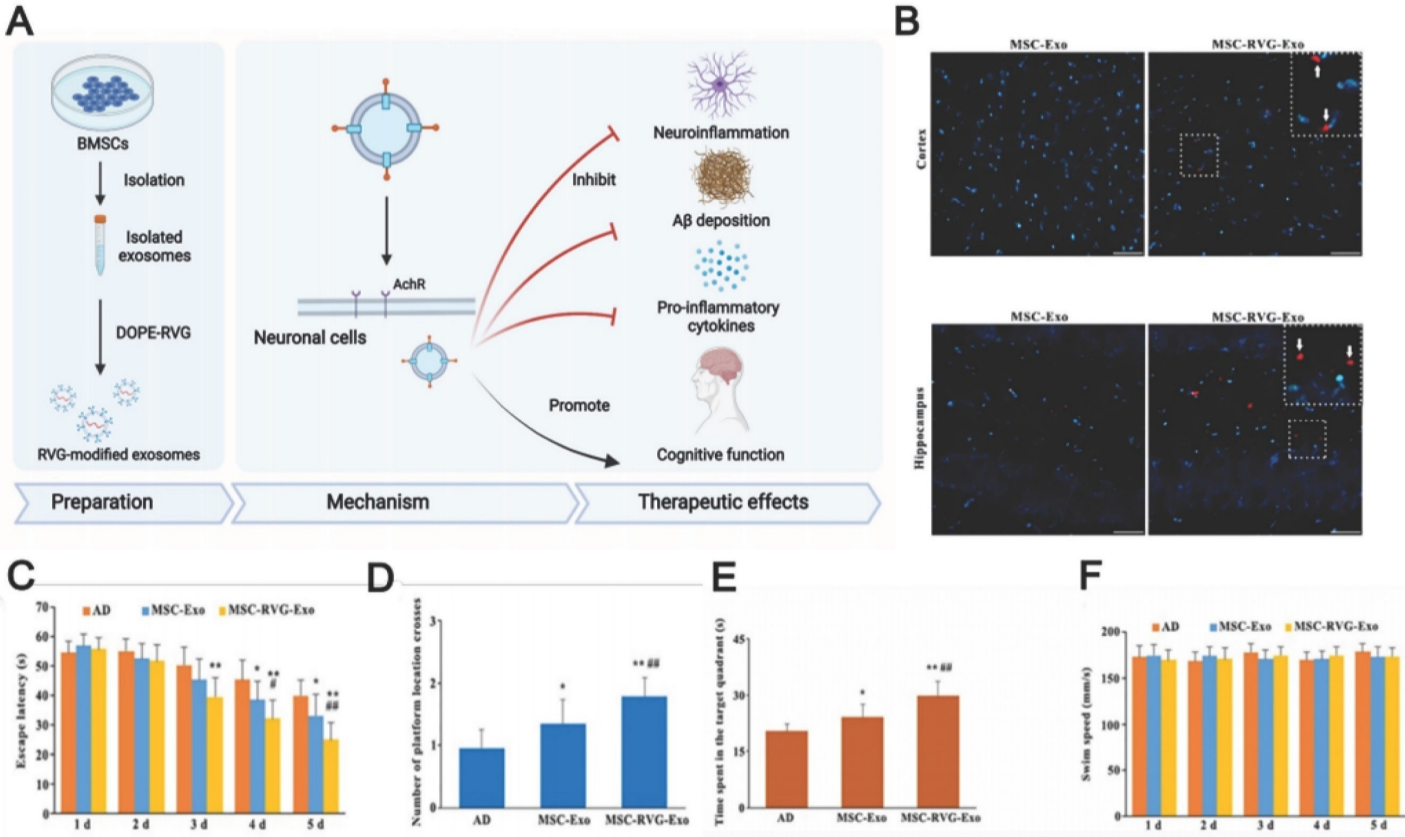
(A) Schematics of the use of RVG-modified MSC-exo for AD therapy. (B) The injected exosomes in the brain. (C, D, E) Morris water maze test results of MSC-RVG-exo and MSC-exo injected AD mice. (F) The swimming speed of the groups. Reproduced from ref. 30. Copyright © 2019 Guo-Hong Cui et al.

Even though MSC-EVs possess native homing properties, innovative technologies providing more specific targeting to drug-loaded EVs remain in high demand. The currently studied EV display technique achieved EV delivery to the brain by facilitating BBB penetration using cell-penetrating peptides such as the rabies virus glycoprotein (RVG), which specifically binds to the acetylcholine receptor or γ-aminobutyric acid (GABA) receptor to enter into neuronal cells through transcytosis. These receptors are widely distributed in microvascular endothelial cells and neurons 164, 165. Cui et al. prepared RVG-decorated EVs by incubating BMSC-EVs with dioleoylphosphatidylethanolamine (DOPE)-RVG obtained by combining RVG and DOPE-*N*-hydroxysuccinimide 30. The resulting MSC-EVs were effective in treating AD in mice, effectively reducing Aβ accumulation, activating microglial cells, and achieving a balanced inflammatory response (Fig. 6). Compared to those treated with non-RVG-tagged exosomes, mice treated with RVG-tagged exosomes exhibited better targeting to the cortex and hippocampus, decreased Aβ protein accumulation, and reduced microglial activation, which highlights the possibility of RVG-modified MSC-EVs in AD treatment. In addition, other peptides, such as those derived from candoxin and brain metastatic breast cancer membranes, have been used to facilitate the entry of red blood cells and polymeric nanoparticles into the brain 166, 167. These peptides may also provide a promising way to enhance MSC-EV penetration. Thus, further experiments are necessary to investigate better methods of enhancing the targeting ability of MSC-EVs through surface modification.

## 6. MSC-EVs for AD therapy in clinical trials

Even though the number of related *in vitro* and animal studies is on the rise, clinical evaluations of MSC-EVs remain limited. EVs derived from allogenic adipose mesenchymal stem cells are among the few EV-based AD therapeutics that have progressed to phase I/II clinical trials (NCT04388982). This study was sponsored by the Ruijin Hospital and involved nine participants divided into three groups that were administered MSC-exos intranasally at different dosages (5, 10, and 15 μg) for 12 weeks. The primary outcome measures involved liver or kidney function and adverse events related to treatment evaluated by CTCAE 4.0 (common terminology criteria for adverse events), while the secondary outcome measures included cognitive function, quality of life, magnetic resonance neuroimaging results, and positron emission computed tomography neuroimaging results. Another outcome that was measured was the change in Aβ levels in serum and CSF. This study has yet to be concluded.

Another clinical study, Focused Ultrasound and Exosomes to Treat Depression, Anxiety, and Dementias (NCT04202770), was chaired by M.D. Sheldon Jordan of the Neurological Associates of West Los Angeles. This study is designed to facilitate the delivery of factors to localised targets using focused transcranial ultrasound before the IV infusion of exosomes derived from healthy full-term caesarean section amniotic fluid. The Quick Dementia Rating Scale is the primary outcome measure, and neuropsychological status and Montreal cognitive assessment results are secondary outcome measures. Starting in December 2019, this study is expected to continue for five years.

The rarity of such studies is ascribed to the need for more robust large-scale production; the ambiguous nature of MSC-EV components; the lack of standardised isolation methods, safety, and doses for clinical use; and methods for MSC-EV characterisation and quantitation.

## 7. Advantages and challenges of MSC-EV use for AD treatment

Along with the ability to penetrate the BBB, MSC-EVs possess properties similar to those of their parents, including immunomodulation, neuroprotection, and active migration 29, and are therefore ideal therapeutics for AD. The following section summarises the advantages and challenges of AD treatment with MSC-EVs.

### 7.1. Advantages

#### 7.1.1. High biocompatibility and low immunogenicity

Compared with MSCs and traditional nanocarriers such as liposomes, MSC-EVs show higher biocompatibility and lower immunogenicity 168, 169. In contrast to MSCs, EVs do not reproduce and undergo uncontrolled division, thus posing a lower risk of tumour formation 170. Furthermore, unlike artificial nanoparticles, which can cause hypersensitivity owing to the inherent immune response toward foreign materials 171, EVs are less immunogenic, less toxic, and more stable in circulation considering their endogenous origin and special surface composition 172. Compared with EVs derived from other cells, MSC-EVs would enhance the useful live and cargo bioavailability of EV-based nanocarriers, owing to the immunomodulatory properties inherited from the parent cells 173.

#### 7.1.2. High efficiency of drug delivery

As highly biocompatible nanoscale materials, MSC-EVs can improve drug absorption and delivery. As natural EVs are capable of surviving hypoxia, they may be crucial to treating diseases characterised by hypoxic tissues, e.g. AD 168. Once injected into the body, nanoparticles are subject to both physical and biological barriers (e.g. shearing force, phagocyte phagocytosis, and renal clearance) before they reach their intended targets. Natural EVs are free from these drawbacks. Moreover, EVs can penetrate the BBB and deliver the cargo into target cells while showing high stability in circulation, making them attractive nanocarriers for AD treatment 174. In addition to intravenous injection, intranasal administration has become an attractive approach with the advantages of rapid onset of action, improved patient compliance, effective brain targeting, and reduced systemic side effects 175. Unlike MSCs, EVs can be stored at -80 °C for up to a year without significant component breakdown 176.

#### 7.1.3. Ease of modification

Bioactive molecules can be loaded into MSC-EVs before or after isolation, as described above. Cell transfection and parental cell incubation with drugs are two main ways of packaging drugs into MSC-EVs 177, allowing the efficient production of EVs loaded with the molecules of interest while preserving the integrity of EV membranes. Drug loading into MSC-EVs after isolation can be achieved in several ways, e.g. hydrophobic drugs can be combined with EVs, while hydrophilic drugs can be made to enter EVs through physical or chemical approaches 178. Drug loading after EV isolation is more effective and significantly increases drug loading capacity 179. Aside from the homing ability, the surface modification of MSC-EVs also increases their ability to penetrate the BBB 180. Moreover, unlike liposomes, which exhibit a low packaging efficiency of hydrophilic cargoes, EVs have a high affinity for nucleic acids, which allows for the more efficient packaging and higher efficiency of these substances 181. Apart from MSCs, immune cells and cancer cells are also popular cell sources for producing EVs. However, EVs derived from immune cells are currently being studied and applied in clinical settings for their antigen-presenting abilities, which are capable for designing vaccination avenues with intrinsically or extrinsically loaded antigens 182. Cancer cell-derived EVs are mostly used in targeted cancer therapy through cancer-associated antigen presentation and have not been reported for AD therapy. Moreover, endogenous oncogenic factors could be carried in cancer cell-derived EVs, contributing to the risk of tumour formulation 183. Hence, MSCs are well suited to mass-producing ideal drug-delivery EVs.

#### 7.1.4. Ease of industrialisation

Although diverse cells can secrete EVs, human MSCs appear to be most promising for commercially sustainable EV production, as they are easily accessible, can be derived from nearly all ethically uncontroversial tissues in the human body and are highly proliferative 184. Besides, The FDA has also approved their use for clinical purposes 84. Thus, MSCs are well suited for producing ideal EVs for drug delivery.

### 7.2. Challenges

#### 7.2.1. Poorly defined therapeutic mechanisms and quality control of MSC-EVs

Though MSC-EVs are rich in various bioactive and functional molecules, their composition is still complex and ambiguous. MSCs secrete detrimental cytokines via paracrine mechanisms, which demonstrate the need to identify EV contents and remove interferences from unknown secretions. It is also essential to determine the source of EVs and consider the differences in the content and therapeutic effect of EVs produced in different culturing environments, such as hypoxic and cytokine-containing environments 185. The EV components are affected by the origin and physiology of parent cells 186. Additionally, MSCs obtained from diverse sources vary in cell yield, survival, and differentiation abilities 187. For example, synaptic structures are generated more efficiently by adipose tissue derived mesenchymal stem cells (AT-MSCs) than by stem cells derived from other sources 188. Furthermore, although EVs exert therapeutic effects on AD, the mechanisms underlying these effects are not completely understood. MSC-EVs are characterised by complex characteristics, making their specification and characterisation difficult due to their limited quality attributes. As a result, apart from specifying and characterising the final product, quality control of raw materials and manufacturing processes is essential for MSC-EVs 189. Therefore, quality and manufacturing controls should be implemented to guarantee EV quality, efficiency, and safety. Therefore, there is a need for further research to pinpoint the exact components of MSC-EVs that play a significant role in AD treatment. Currently, no research exists that compares the components of EVs from different MSCs, and differences in the therapeutic efficacy of such EVs are still unclear. To further elucidate the mechanism of MSC-EVs in AD therapy, one should identify the exact composition of EVs using multiomics methods.

Particle and protein concentrations differ according to the experimental methods, making comparisons more difficult 190. Researchers found large inconsistencies in the doses used in 64 preclinical studies across several disease areas. They suggested adopting the parameters of therapeutic effects rather than quantification in EV dose determination 191.

While EV therapy was used for the first time in 2005 for cancer treatment 192, no standard methods have been established for quality control of EVs for clinical use. Strategies like ELISA, aptamer‐ and carbon nanotube‐based colourimetric assays, and bead‐based flow cytometry can be used to quantify the specific cargoes in EV products 193. For reproducibility and quality assurance of MSC-EVs, reliable quality control of the contained particles must be established. For example, the minimal information for studies of EV (MISEV) recommends showing at least one transmembrane protein or extracellular lipid-bound protein on EVs, initially developed to identify EVs.

#### 7.2.2. Complicated procedures

EV separation methods have inherent advantages and disadvantages, and there is no consensus regarding the best isolation approach. Moreover, the protein and RNA contents of EVs significantly depend on the isolation method 194. Better production methods for MSC-EVs are required to facilitate their clinical use. Recent studies have demonstrated that size-exclusion chromatography is more effective at separating MSC-EVs than differential centrifugation. However, the limitation still involves labour-intensive processes and does not ensure the complete clearing of co-contamination with proteins and lipoproteins 195. Considering their small yield and physicochemical heterogeneity, MSC-EVs are challenging to isolate in high yield or purity. Therefore, simpler, inexpensive, and mass-production-suitable methods should be developed to step forward in the clinical research and application of MSC-EVs. Moreover, no standard method currently exists for EV isolation. Thus, MSC-EV therapeutics need further regulatory oversight before they can be safely translated from the bench to the bedside 196.

## 8. Concluding remarks and outlook

Ninety-eight per cent of small molecules and almost 100% of large molecules are unable to penetrate through the BBB, despite a substantial number of neurotherapeutics being developed in different disease models. Due to their natural tendency to cross the BBB and their therapeutic potential, MSC-EVs have attracted attention as a new-generation drug delivery system for AD therapy. Despite such potential, challenges remain, such as low targeting efficiency, nonuniform treatment results, and low production yield. Therefore, advanced engineering techniques are needed to functionalise MSC-EVs and thus increase their efficiency and potency as AD therapeutics.

**Figure 7:**
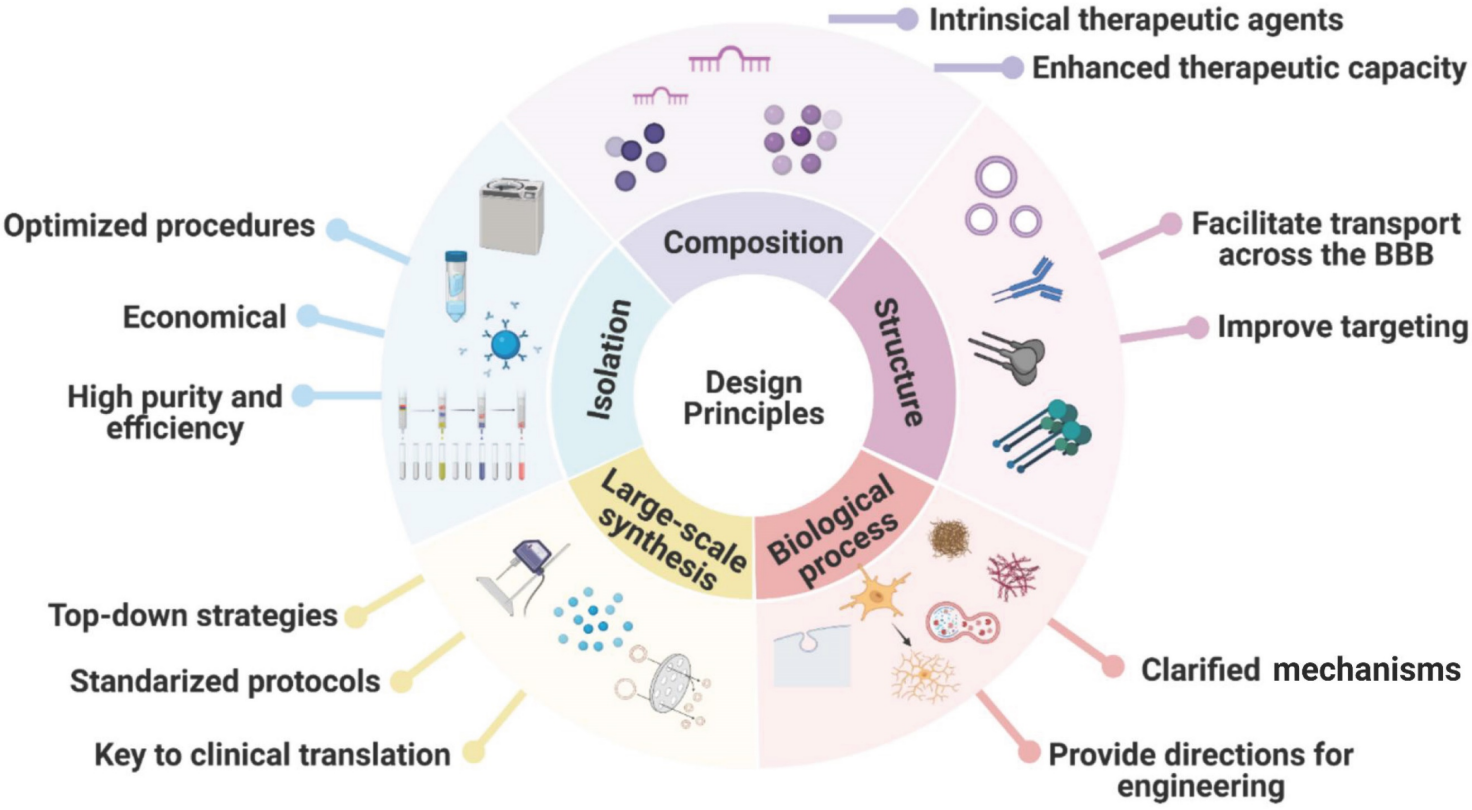
Design principles for engineered MSC-EVs as nanocarriers for future clinical application.

MSC-EVs exhibit many advantages, e.g. high biocompatibility, low immunogenicity, high drug delivery efficiency, ease of modification, and ease of industrialisation, and can alleviate AD symptoms by releasing loaded therapeutic agents to promote Aβ degradation, immunomodulation, and neuroprotection. Because AD pathology is unclear and considered to be multifactorial, MSC-EVs serve as a therapy that can target various pathogeneses of AD. In this review, we have summarised the advances in the use of MSC-EVs for AD treatment, focusing on naturally secreted MSC-EVs, bioengineered MSC-EVs, and functionalisation strategies while highlighting the encouraging preclinical results reported recently. The design principles for engineered MSC-EVs for future clinical application are summarised in Figure 7.

Developing EV-based therapies for clinical use is hampered by the limited availability of large-scale well manufactured EV production. Currently, the 2D culture method is the most used strategy for large-scale EV production, which is restricted by cell cloning, differentiation, and expansion 197. In comparison to this method, approximately 40 times more EVs are produced by hollow fibre bioreactor cultures, which may serve as an alternative culture method to obtain EVs on a large scale. 198. Despite this, 3D cell culture still has limitations. It is difficult to manually enlarge hanging objects using the 3D culture method and because exosome production efficiency depends on sphere size, production stability is inconsistent 199.

New drug delivery platforms based on MSC-EVs have recently been developed. The three main ways of adding the desired functionality to MSC-EVs are the enhancement of MSC-EV contents by the preconditioning of parent cells, EV surface modification for specific delivery of therapeutic cargoes, and EV loading with exogenous cargoes. Using artificial EVs allows one to conquer the disadvantages of natural EVs, including low productivity and tedious isolation methods, even though artificial EVs have not been used in AD therapy. The combination of MSC-derived membranes with nanomaterials featuring unique physical/chemical properties is also a promising solution for future AD therapy, catalysing the development of innovative biomaterials that possess multiple functions, high biocompatibility, and negligible immunogenicity for AD treatment. We expect this review to raise awareness concerning the potential of MSC-EVs as efficient nanocarriers for AD treatment, in addition to exhibiting an inherent therapeutic effect.

Even with the active progress in MSC-EV usage in AD therapy, many challenges, including those associated with isolation, yield, and standardisation processes, remain to be overcome before the related clinical translation. The large-scale production of MSC-EVs with acceptable batch-to-batch variation is challenging because of their heterogeneity, which is influenced by the culturing environment and the MSC source. To ensure a safe and effective MSC-EV therapy, it is important to select the right cell source. When using autologous cells, mismatched antigens are avoided, reducing the risk of host immune responses. Conversely, allogenic (donor) cells can provide readily available cell sources when needed. Nonetheless, in an analogous way to organ transplantation, antigen matching is essential for avoiding host immune responses. Consequently, MSC-EVs should be produced on a large scale with stringent quality control. Building donor cell banks that are easily accessible for the selection of proper cell sources may be an alternative approach for future clinical applications. Gene-editing techniques may also facilitate the deletion of unwanted immunogenic proteins by deleting specific genes. The use of CRISPR/Cas9 and zinc finger nuclease, for example, has demonstrated the feasibility of removing human leukocyte antigen genes and generating more immune-compatible stem cells 200, 201. Long-term treatments could be achieved by MSC-EVs derived from these modified cells. Additionally, quality control standards and normative methods of isolation and characterisation should be established. Despite these challenges, MSC-EVs still represent a potential multifunctional weapon for AD therapy owing to their unique characteristics and biofunctions.

## Figures and Tables

**Table 1:** Overview of methods used for EV isolation.

Methods	Principle	Advantages	Disadvantages	References
Differential ultracentrifugation	Differential Centrifugation based on density	High purity; Established protocols	Lengthy process; Large sample Volume	39
Gradient density ultracentrifugation	Density additional steps after centrifugation	High purity; Avoiding exosomal damage	Labour-intensive; Preliminary preparation; Cumbersome operation	40
Ultrafiltration	Based on particle size and molecular weight	High yields; High purity; Requires no special equipment; Easy to handle	Contamination of same-sized vesicles; Low specificity	41
Size-exclusion chromatography	Based on hydrodynamic Radius	Better repeatability; Time efficient; Less protein contamination	Low sample recovery	42
Immunoaffinity	Based on antibodies' interactions with specific membrane proteins in EVs	High specificity for EV subtypes isolation	High cost; marker dependent	43
Polymer-Based Precipitation	Precipitation with chemicals	Simple; fast	Lack specificity; much contamination; difficulty in scaling	44
Microfluidics-Based Isolation	Based on physical or mechanical properties	Fast; cheap; require less volume	Unsuitable for large samples; expensive equipment support	45

**Table 2:** The surface markers of MSC-EVs, storage, and routes of administration in disease treatment.

EV source	Surface markers	Storage	Route of administration	References
hBMSCs	CD90, CD44, CD105, CD73, CD63, CD81, neuropilin 1 (+) CD34, CD45 (-)	storage at -80℃ in PBS with or without cryoprotective additives	lateral ventricle injection, IV, IN	58-62
hCMSCs	CD105 (+), CD90 (low), CD117 (-)	storage at -80℃ in PBS with or without cryoprotective additives	ND	62, 63
hUC-MSCs	CD90, CD73, CD105, CD44, CD9, CD63, CD81 (+) CD45, CD34, CD11b, CD19, HLA-DR (-)	storage at -80℃ in PBS with or without cryoprotective additives	IV	62, 64, 65

hBMSCs, human bone marrow-derived mesenchymal stem cells; hCMSCs, human cardiac mesenchymal stromal cells; hUC-MSCs, human umbilical cord-derived MSCs; PBS, Phosphate‐buffered saline.

**Table 3:** Effects of native MSC-EVs on AD in animal models

	EV Source	Route of Administration	Therapeutic Effects	Mechanism of Action	References
In vivo					
	rBMSCs (Primary cell)	lateral ventricle injection	↓Aβ deposition and soluble Aβ_1-42_ ↑Neuron viability ↓Neuron apoptosis rate ↓Pro-inflammatory factors (IL-1β, IL-6, and TNF-α)	↑Expressions of NEP and IDE ↑Expression of miR-29c-3p ↓Expression of BACE1 ↑Activation of Wnt/β-catenin pathway	97
	mBMSCs (Primary cell)	IV	↓Aβ deposition ↑Cognitive function	↑Activation of SphK/S1P	98
	WJM-SCs (Primary cell)	IV	↓Aβ deposition and astrocyte activation ↑Brain glucose metabolism and cognitive function	↓Expression of HDAC4 ↑Expression of neuronal memory and synaptic plasticity-related genes	32
	hUCMSCs (Primary cell)	IV	↓Immune memory in the brain ↓Deteriorated neuropathology caused by immune training	IL-10 dependent mechanism	88
	hUCMSCs (Primary cell)	IV	↓Aβ deposition, neuronal loss and calcium transients ↑Cognitive function and hippocampal neuronal excitability Neuronal morphology alterations repair ↓mitochondrial changes	↓Associated with Nrf2 defence system Nrf2, HO-1, iNOS, NQO1 ↑Keap1	31
	mBMSCs (Primary cell)	lateral ventricle injection	↑Cognitive function	↑CA1 synaptic transmission ↓LTP and iNOS expression	92
	mBMSCs (Primary cell)	lateral ventricle injection/IV	↑Cognitive function ↓Activation of microglia and astrocytes ↓IL-1β, IL-6, TNF-α, Aβ1-42, p-Tau	↑Expression of BDNF	99
	hBMSCs (Primary cell)	Intracerebroventricular CSF exchange	↑Cognitive function and neuronal counts ↓Astrocytic burden ↑Cell proliferation and neurogenesis	ND	90
	rBMSCs (Primary cell)	IV	Improves the degenerative effects on circumvallate taste buds and gustatory nerve fibres	ND	91
*in vitro*					
					
	hUCMSCs (Primary cell)		↓Apoptosis rate and concentrations of inflammatory factors	↑Expression of miR-223 Inhibition of PTEN-PI3K/Akt pathway	89
	hADSCs (Primary cell)		↓ Extracellular and intracellular Aβ levels	NEP associated mechanism	100

BDNF, brain-derived neurotrophic factor; CSF, cerebrospinal fluid; hADSCs, human adipose tissue-derived mesenchymal stem cells; hUCMSCs, human umbilical cord tissue-derived mesenchymal stem cells; hBMSCs, human bone marrow-derived mesenchymal stem cells; IV, intravenous injection; LTP, long-term potentiation; mBMSCs, mouse bone marrow-derived mesenchymal stem cells; rBMSCs, rat bone marrow-derived mesenchymal stem cells; WJ-MSCs, Wharton's jelly-derived mesenchymal stem cells; ND, not detected.

**Table 4:** Applications of engineered MSC-EVs in AD treatment.

EV Source	Modification	Route of Administration	Therapeutic Effects	Mechanism of Action	References
mBMSCs (Primary cells)	Surface Modification with RVG peptide	IV	↑Learning and memory function	↓Aβ deposition and activation of astrocytes ↓Pro-inflammatory factors ↑Anti-inflammatory factors	30
rBMSCs (ND)	Loaded miR-29	dorsal hippocampus injection	↑Cognitive and learning function	↓Expression of BACE1 ↑Activation of PKA/CREB	56
mADSCs (ND)	Loaded miR-22	IV/coculture	↑Cognitive and learning function	↓Pro-inflammatory factors ↓Neuroinflammation ↓Damage to nerve cells ↓Inflammation and pyroptosis ↓Expression of NLRP3, GSDMD and p30‐GSDMD	155
hUCMSCs (Primary cells)	Loaded NEP	IV	↑Memory function	↓Aβ deposition and inflammatory Response ↓Expression of BACE-1 ↑Expression of NEP ↑BDNF levels and neurogenesis	65
mBMSCs (Primary cells)	preconditioned *in vitro* in an AD environment	IV/IN	**↑Memory function and lifespan**	↓Aβ deposition and AβO concentration ↓Neuroinflammation ↑Neuronal density	76
mADSCs (Primary cells)	hypoxic preconditioned	ND	↑Cognitive function	↓Nerve apoptosis Circ-Epc1 associated mechanism ↓Expression of miR-770-3p and inflammatory factors ↑Expression of TREM2 Shifting microglial M1/M2 polarisation	156
mBMSCs (Primary cells)	hypoxic preconditioned	IV	↑learning function ↑Memory function	↓Aβ deposition and pro-inflammatory factors ↑Expression of synapsin 1 and PSD95 ↓Activation of astrocytes and microglia ↓Shifting microglial M1/M2 polarisation ↑Anti-inflammatory factors and level of miR-21 ↓Phosphorylation of STAT3 and NF-kB activation	57
hBMSCs (Primary cells)	TNFα and INFγ -preconditioned	IN	ND	↓Shift microglial M1/M2 polarisation, microglia activation ↑Dendritic spine density	58
hUCB-MSC (Primary cells)	Graphene scaffold 3D culture	Coculture/hippocampus injection	↑Learning and Memory Function	↑Expression of NEP, IDE, HSP70 ↓Aβ deposition, TNF-α, IL1β	157

GSDMD, gasdermin D; IN, intranasal administration; IV, intravenous injection; hBMSC, human bone marrow-derived mesenchymal stem cells; hUCBMSC, human umbilical cord blood-derived mesenchymal stem cells; hUCMSCs, human umbilical cord tissue-derived mesenchymal stem cells; mADSCs, mouse adipose-derived stem cells; mBMSCs, mouse bone marrow-derived mesenchymal stem cells; NEP, neprilysin; NLRP3, Nucleotide oligomerization domain-like receptor thermal protein domain associated protein 3; rBMSCs, rat bone marrow-derived mesenchymal stem cells; RVG, rabies viral glycoprotein; TNF, tumor necrosis factor; ND, not determined.

## Abbreviations

AβO): Aβ oligomer
APP): Aβ precursor protein
ADSC): adipose tissue stem cell
AD): Alzheimer's disease
Aβ): amyloid-β
BACE1): β-site amyloid precursor protein cleaving enzyme 1
BIM): Bcl-2 interacting mediator of cell death
BACE): β-site APP-cleaving enzyme
BBB): blood-brain barrier
BDNF): brain-derived neurotrophic factor
BMSC-EV): bone marrow mesenchymal stem cell-derived extracellular vesicle
BMEC): brain microvascular endothelial cell
BMEC): brain microvascular endothelial cell
CNS): central nervous system
CSF): cerebrospinal fluid
DOPE): dioleoylphosphatidylethanolamine
EV): extracellular vesicle
GSDMD): gasdermin D
hADSCs): human adipose tissue-derived mesenchymal stem cells
hBMSCs): human bone marrow-derived mesenchymal stem cells
hUCMSCs): human umbilical cord tissue-derived mesenchymal stem cells
hUCBMSCs): human umbilical cord blood-derived mesenchymal stem cells
HIF-1α): hypoxia-inducible factor
ILV): intraluminal vesicle
IN): intranasal administration
IV): intravenous injection
LTP): long-term potentiation
mADSCs): mouse adipose-derived stem cells
mBMSCs): mouse bone marrow-derived mesenchymal stem cells
MSC): mesenchymal stem cell
MSC-EV): mesenchymal stem cell-derived extracellular vesicle
MVBs): multivesicular bodies
NLRP3): NOD-like receptor thermal protein domain associated protein 3
ND): not detected
NEP): neprilysin
NF): neurotropic factor
PTEN-PI3K/Akt): phosphatase and tensin homolog-phosphatidylinositol-3-kinase
PBS): Phosphate‐buffered saline
RVG): rabies viral glycoprotein
rBMSCs): rat bone marrow-derived mesenchymal stem cells
SphK): sphingosine kinase
S1P): sphingosine 1-phosphate
SHP2): Src homology 2 domain-containing protein tyrosine phosphatase-2
GABA): Wharton's jelly-derived mesenchymal stem cells (WJ-MSCs
